# Progress of Inertial Microfluidics in Principle and Application

**DOI:** 10.3390/s18061762

**Published:** 2018-06-01

**Authors:** Yixing Gou, Yixuan Jia, Peng Wang, Changku Sun

**Affiliations:** 1 State Key Laboratory of Precision Measurement Technology and Instruments, Tianjin University, Tianjin 300072, China; gouyx@tju.edu.cn (Y.G.); sunck@tju.edu.cn (C.S.); 2 Department of Biomedical Engineering, Tsinghua University, Beijing 100084, China; jia-yx14@mails.tsinghua.edu.cn

**Keywords:** inertial microfluidics, Dean vortex, particle manipulation, lab-on-a-chip

## Abstract

Inertial microfluidics has become a popular topic in microfluidics research for its good performance in particle manipulation and its advantages of simple structure, high throughput, and freedom from an external field. Compared with traditional microfluidic devices, the flow field in inertial microfluidics is between Stokes state and turbulence, whereas the flow is still regarded as laminar. However, many mechanical effects induced by the inertial effect are difficult to observe in traditional microfluidics, making particle motion analysis in inertial microfluidics more complicated. In recent years, the inertial migration effect in straight and curved channels has been explored theoretically and experimentally to realize on-chip manipulation with extensive applications from the ordinary manipulation of particles to biochemical analysis. In this review, the latest theoretical achievements and force analyses of inertial microfluidics and its development process are introduced, and its applications in circulating tumor cells, exosomes, DNA, and other biological particles are summarized. Finally, the future development of inertial microfluidics is discussed. Owing to its special advantages in particle manipulation, inertial microfluidics will play a more important role in integrated biochips and biomolecule analysis.

## 1. Introduction

Cells and biomolecules such as proteins, nucleic acids, and exosomes are the fundamental units of life. Their behaviors and characteristics determine the functions and status of tissues and organs [1]. Detailed analysis on such biomolecules is of great significance in biological studies and clinical diagnosis [2,3]. The pre-treatment of biomolecules through microfluidics, such as focusing [4,5], patterning [6], separation [7], mixing [8], and enrichment [9,10], has been considerably developed and plays important roles in the research areas of life science and biomedicine. Among these operations, i.e., focusing and separation, are the most important pre-treatments in biomedical examinations. Focusing is used in the preprocessing of on-chip microfluidic flow cytometers. Separation is applied to separate the biomolecules (circulating tumor cell, droplet, exosomes, and bacteria) through their inherent qualities, such as size [11,12], dielectric property [13,14], optical or image characteristics [15,16,17], and biological immune characteristics [18,19].

Inertial microfluidics is an extension of conventional macroscopic inertial fluidics methods to the unconventional micro/nano scale. Though lacking in specificity, inertial microfluidics has been widely investigated in the manipulation of particles such as focusing, ranking, separation, transfer, and mixture, which have been demonstrated in the recent past [20].

Compared with conventional microfluidics, inertial microfluidics is superior. First, the operation is easy, as the target can be realized only by adjusting the flow velocity of the sample. Second, the channel structure is simple. Therefore, extra external fields and other methods can be easily integrated on the microfluidics chip, thus reducing the cost of the product. Third, compared with other methods, the throughput is extremely high, so an experiment can be completed in a very short time. Finally, inertial microfluidics has little influence on the activities of biomolecules, so it is suitable for biomedical devices. Owing to these advantages, inertial microfluidics is applied to manipulate biological particles for many purposes, such as the acquisition of circulating tumor cells (CTC), droplet packages of single cells, removal of pathogenic bacteria, detection of exosomes, and DNA focusing.

## 2. Principle of Manipulation by Inertial Microfluidics

In traditional microfluidic devices, the Reynolds number of the flow field is low, and the flow is regarded as Stokes flow [21]. The inertial effect of the flow field and particles can be ignored. Compared with traditional microfluidic devices, the flow field in inertial microfluidics is between the Stokes state and turbulence, whereas the flow is still regarded as laminar. However, the inertial and viscosity effects will also affect the movement of particles, thus making the manipulation of particles more complicated.

### 2.1. Theory of Inertial Effect in Straight Channels

The inertial effect was first proposed by Segre and Silberberg in *Nature* in 1961 [22]. It was observed in a macroscopic pipe where millimeter-sized suspended particles that were initially randomly distributed in the circular tube (~1 cm) migrated laterally to focus on an annulus with a radius of 0.6 times the radius from the center of the pipe. It was shown that when the particles flow in the flow tube with a low Reynolds number, apart from the mainstream driving force along the mainstream direction, there was also a lateral lift force perpendicular to the mainstream, eventually leading to a lateral migration to the dynamic equilibrium position [23]. This phenomenon aroused the attention of many scholars, and many achievements were made in the study of the inertial focusing effect in different section shapes [24].

**Figure 1:**
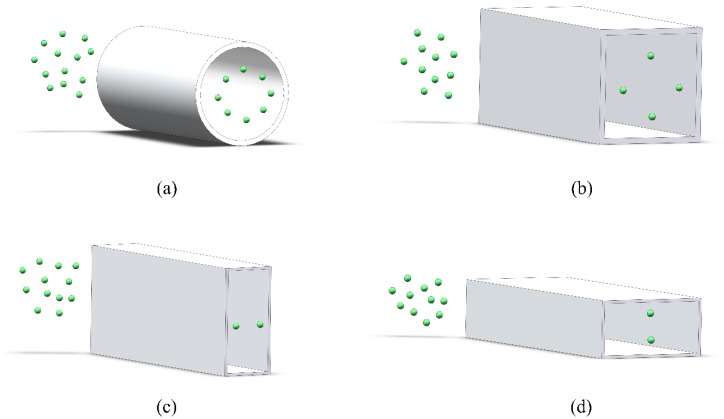
Schematic of the focusing position of particles migrating through channels with different cross-section shapes: (**a**) circular channel; (**b**) square channel; (**c**) high-aspect-ratio rectangular channel; and (**d**) low-aspect-ratio rectangular channel.

The final equilibrium position of particles depends on the channel section. In the medium Reynolds-number condition, the particles migrating in a circular channel form an annulus that is called the Segre-Silberberg annulus [25] (Figure 1a) because of the symmetry of the circle. As for a square section, owing to the influence of the edge angle on the velocity and pressure distribution, an offset correction of the equilibrium position will occur so that the particles migrating in a square-section channel are located in the four equilibrium positions close to the midpoint of the channel wall (Figure 1b). In a high/low-aspect-ratio square section, the shear gradient in the short channel wall is far greater than that of the long channel wall. Therefore, the unstable focus position of the short wall will be revised to the center of the long wall. In a high/low-aspect-ratio rectangular microchannel, the particles finally flow to the two equilibrium positions close to the midpoint of the long channel wall (Figure 1c,d) [26].

There are also many groups that are studying the focusing position and morphology of the particles in different conditions [27]. Liu [28] conducted 3D direct numerical simulations (DNS) and found that apart from the two focusing positions near the long channel walls, there are additional stable equilibrium positions close to the short walls. Di Carlo [29] showed that the value of *a*/*H* should be known to determine the equilibrium position. B. Chun [30] presented numerical evidence that particles can migrate to the center at a high Reynolds number (700–1000) through the formation of hydrodynamic clusters. At present, for the three-dimensional dynamic process of particles, the most common mathematical model is the two-stage model proposed by Zhou [31]. This process can be summarized as follows: In stage 1, in the condition of a moderate Reynolds number, the particle migrates to the equilibrium position close to the wall under the shear-induced inertial lift force and wall-induced inertial lift force, and in stage 2, the particles continue to migrate to the equilibrium position at the center of the wall under the effect of a spin-induced Saffman lift force. Moloudi [32] studied particle focusing dynamics inside trapezoidal straight microchannels and found the lateral focusing depends on the particle clogging ratio, channel aspect ratio, and slope pf slanted wall. The particle focusing principle in the microchannel, with the cross-sectional shape of the isosceles right triangle, is also studied by Kim [33].

### 2.2. Theory of Inertial Effect in Curved Channels

**Figure 2:**
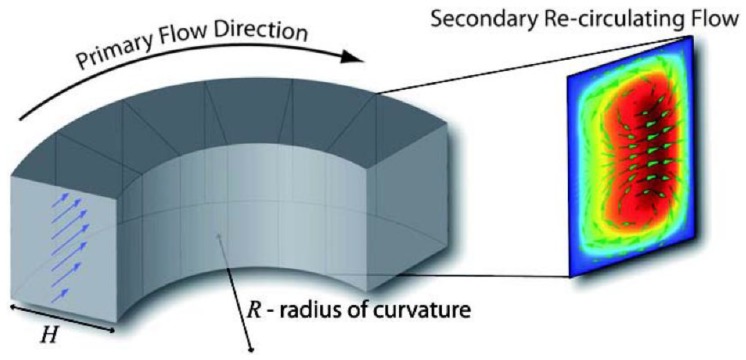
Secondary flow in cross-section. Ref. [40]. Copyright (2009), with permission from the Royal Society of Chemistry.

In a curved channel, the velocity profile of the Poiseuille flow in the main flow direction appears to be parabolic, and the fluid in the central area has a higher velocity than that in the wall area. Therefore, when the particle goes through the curved channel, it flows from the central line to the outward channel because of the inertia, leading to an imbalance in the radial pressure gradient. By contrast, in a closed channel, to meet mass conservation, the fluid near the outward wall recirculates inward, owing to the centrifugal pressure gradient. Two vortices with opposite directions of rotation are produced in the cross-section of the vertical direction (shown in Figure 2). This phenomenon is called the cross-section secondary flow. Its characteristics are represented by a dimensionless coefficient *D_e_*. This coefficient was first proposed in the research of fluid motion in a curved channel by Dean [34,35]. Berger presented a calculated mode of the Dean number, which has been widely used so far. The expression of the Dean number is as follows:

(1) $$D_{e}=R_{e}\sqrt{\frac{H}{2R}}$$

where *R* is the curvature radius of the channel, and *H* is the hydraulic diameter. With an increase in *R_e_*, the Dean flow will also increase. In addition, the shape of the Dean flow is also related to the *D_e_* number. With an increase in *D_e_*, the center of the Dean vortex moves toward the outer wall, and the number of the Dean flow also changes at high velocity. In general, the *D_e_* number in a straight channel is zero, which means that there is no Dean flow in a straight channel in the laminar flow state. The velocity of the secondary flow in a Dean vortex is another important parameter that characterizes the Dean flow. This parameter was given by Ookawara [36] through a numerical simulation in which $U_{D}=1.8\times 10^{-4}D_{e}^{1.63}$. This expression is widely used in the estimation of applied research [37]. Recently, Dinler [38] modelled and simulated the hydrodynamic forces acting on the particle under the effect of Dean vortex, and then predicted the width of the focusing band for different-sized particles. Their simulations are validated with available experimental data. Xiang [39] explored the elasto-inertial focusing behaviors of particles in a spiral microfluidic channel and first proposed a six-stage process model illustrating the particle focusing process in Dean-coupled elasto-in-ertial flow with increasing flow rate.

The Dean flow in particle migration has two main effects: (1) The Dean flow can bring a stirring effect that can accelerate the lateral displacement of the particles, making it faster to migrate to the equilibrium position. Compared with the inertial focusing effect in a straight channel, the length of the focusing channel is greatly shortened because of the Dean flow [41]; and (2) The existence of the Dean flow will influence the distribution of the equilibrium position of the particles in the section of the channel.

### 2.3. Other Factors Affecting the Inertial Effect

As we all know, the most important factor that affects the inertial effect is the cross-section of the channel, but there are other factors that affect the final equilibrium position of particles, including the velocity of the flow, viscosity, deformation, and particle densities.

**Figure 3:**
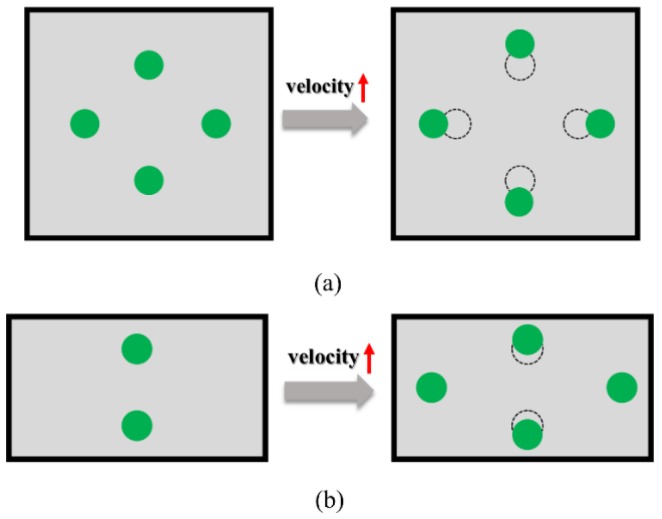
Distribution of equilibrium positions with an increase in velocity in different cross-sections: (**a**) square channel and (**b**) rectangular channel.

The distribution of the inertial equilibrium position of the particle is closely related to the velocity of the particle. In a square channel, with an increase in the velocity, the four inertial equilibrium positions migrate closer to the channel, which is shown in Figure 3a. By contrast, for a rectangular channel, with an increase in the velocity, the two unstable positions at the center of the short wall will gradually stabilize, and four equilibrium positions will be formed (Figure 3b).

When a polymer is added to the Newton fluid, the viscoelastic-induced force effect in the mixed fluid modifies the equilibrium position of the particle [42]. Under the effect of the viscoelastic force and the inertial lift force, the particle migrates to the equilibrium position in the middle line of the channel. Therefore, a polymer is often added to the solution to generate a single equilibrium position in the straight channel [43]. Apart from straight channels, this effect was also used in curved channels and was gradually developed for particle separation methods using the Dean drag force and inertial effect [44]. Xiang [5] proposed a strategy for controlling the particle focusing position in Dean-coupled elasto-inertial flows through adjusting the polymer concentration of viscoelastic fluids.

At present, rigid spheres are often used as basic models for inertial microfluidics analyses. However, real particle samples are usually deformable items such as cells and exosomes. The deformation of the particle will also affect the lateral migration of the particles and impose an extra lift force on them, namely, a deformation-induced lift force. Although the mechanism of the lift has not been fully studied, it is widely accepted that this lift is caused by the matching of velocity and stress at the interface of deformable particles. A circulating tumor cell separation structure based on this model was developed [45].

As the manipulation objects of the inertial microfluidics are usually cells, their density is close to that of the liquid environment, making the inertial centrifugal force ignorable. However, the density difference between a particle and the surrounding liquid also affects the equilibrium positions of the particles [36]. The equivalent centrifugal force can be expressed as

(2) $$F_{C}=\left(\rho_{p}-\rho_{f}\right)\pi V_{pt}^{2}a^{3}/6r$$

where $\rho_{p}$ is the density of the particle, $\rho_{f}$ is the density of the fluid, $V_{pt}$ is the tangential rate of the particles, *a* is the diameter of the particle, and *r* is the curvature radius of the particle trajectory. When there is a difference between the particle density and fluid density, the influence of the centrifugal force on the lateral migration trajectory cannot be ignored.

### 2.4. Force Analyses of Particles in Inertial Microfluidics

In inertial microfluidics, there are nonlinear forces, including the axial drag and lateral force in different forms, that can be used to manipulate particles. The combination of these forces makes the trajectories of the particles in the microchannel more complex.

#### 2.4.1. Inertial Lift Force

When particles migrate in the microchannel, apart from the drag effect, which makes the particles accelerate to the same velocity along the main flow direction as the surrounding fluid, there is also lateral migration perpendicular to the main flow direction. Therefore, the particles will eventually migrate to the equilibrium position [46]. The force that leads to the lateral migration is called the inertial lift force (*F_L_*). In microfluidic chips, the particles in the straight channel are subjected to gravity and buoyancy, which are distributed in the vertical direction, having almost no influence on the lateral migration. Thus, the effect of gravity and buoyancy on particle migration is negligible. There are also other forces that are far weaker than the inertial lift force whose influence on the lateral migration is negligible. Therefore, the only force that leads to the lateral migration of particles is the inertial lift force [47,48].

**Figure 4:**
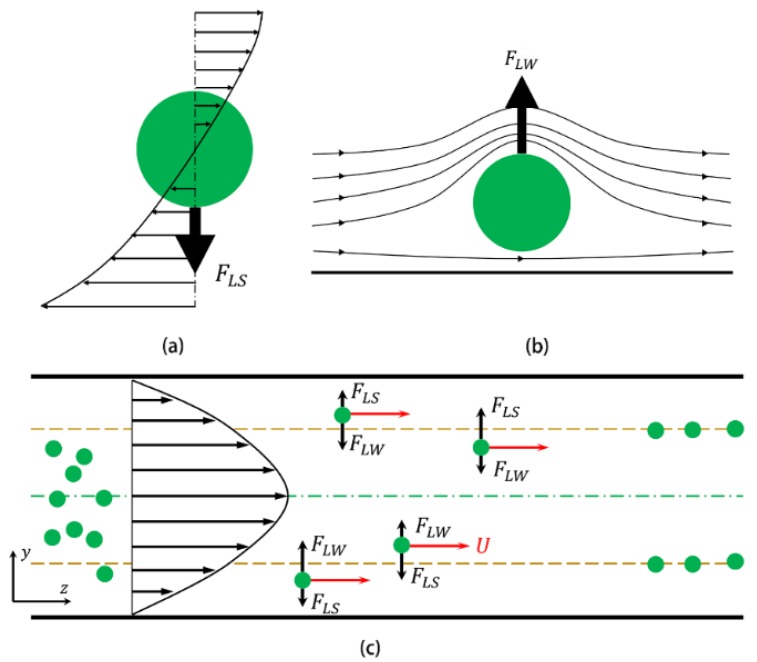
(**a**) Flow field of *F_LS_*; (**b**) Flow field of *F_LW_*; (**c**) Flow field distribution in channel and inertial-lift-force schematic diagram.

Later theoretical analyses showed that there are two types of main lift forces that compose most of the inertial lift force [49]. The velocity profile of a Poiseuille flow in a microchannel appears to be parabolic, leading to a shear-induced inertial lift on the particle, whose direction is from the center of the flow to the wall. This is called the shear-gradient-induced lift force (*F_LS_*), which has the expression $F_{LS}\propto \rho U^{2}a^{3}/H$, where *ρ* is the density of the fluid, *U* is the velocity of the fluid, *a* is the diameter of the particle, and *H* is the hydraulic diameter. The flow field of *F_LS_* is shown in Figure 4a. In addition, owing to the rotation of particles in the fluid, there is a symmetric wake around them. When particles migrate close to the wall, the symmetry of the wake is affected by the wall, inducing a force to drive the particles away from the wall. This is called the wall-induced lift force (*F_LW_*), which has the expression $F_{LW}\propto \rho U^{2}a^{6}/H^{4}$. The flow field of F_LW_ is shown in Figure 4b. These two forces are not fixed but are changeable along the position of the particles in the channel. When a particle migrates close to the central line of the channel, the shear-gradient-induced lift force is dominant. However, when a particle migrates close to the wall, the wall-induced lift force is dominant [50]. The equilibrium position is the point where the shear-gradient-induced lift force equals the wall-induced lift force. The flow field distribution in the channel and an inertial-lift-force schematic are shown in Figure 4c.

Through a study of the distribution of the Poiseuille flow and an analysis of the various influencing factors, the expression of the inertial lift force is [41]

(3) $$F_{L}=f_{L}\left(R_{e},x/h\right)\cdot \rho U^{2}a^{4}/H^{2}$$

where *U* is the maximum velocity of the fluid, which can be estimated as twice the average characteristic velocity. *H* is the hydraulic diameter and is regarded as H=D in a circular channel, where *D* is the diameter of the circular cross-section, or H=2wh/(w+h) in a rectangular channel, where *w* and *h* correspond to the width and height of the rectangular cross-section, respectively. *f_L_* is a nondimensional lift coefficient whose value depends on the Reynolds number of the channel and the specific position of the particle in the cross-section. Previous research found that the lift coefficient decreases with an increase in *R_e_*. When it is applied in the microchannel, the coefficient is regionally dependent, and the lift coefficient near the wall decreases with the increase in *R_e_*, while in the area near the center of the channel, the coefficient increases with the increase in *R_e_*. However, regarding practical microfluidic applications, the lift coefficient can be estimated as $f_{L}=0.5$.

#### 2.4.2. Dean Drag Force

**Figure 5:**
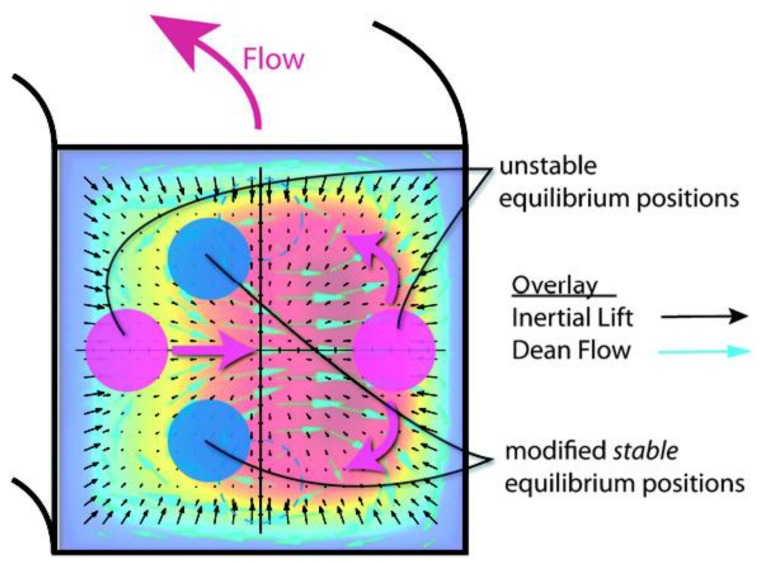
Superposition of effects of inertial migration and secondary flow on particles in curved channel cross-section. Ref. [40]. Copyright (2009), with permission from Royal Society of Chemistry.

Owing to the existence of the secondary flow, the particles in a curved channel are also affected by a Dean drag force, which is expressed as $F_{D}\propto \rho U^{2}a_{p}H^{2}R^{-1}$, where *ρ* is the density of the fluid, *U* is the velocity of the fluid, *a_p_* is the diameter of the particle, *H* is the hydraulic diameter, and *R* is the curvature radius of the channel. In addition, the Dean drag force can also be assumed as the Stokes drag force:

(4) $$F_{Stokes}=3\pi \mu a_{P}U_{D}$$

where *μ* is the fluid viscosity, *a_p_* is the diameter of the particle, and *U_D_* is the Dean flow velocity. In fact, the magnitude and direction of the force depend on the intensity and direction of the Dean flow velocity field in the cross-section. In a curved channel, the movement of particles depends on the superposition of the inertial migration effect and the secondary flow on the particle. This is shown in Figure 5. Gossett proposed that at a low velocity, a particle will be subjected to the interaction of the lateral inertial lift force and Dean drag force [51].

In a curved channel, *R_f_*, the ratio of the lateral inertial lift force to the Dean drag force, is an important parameter that determines the specific characteristics of the particle. When *R_f_* is close to zero, the inertial lift force is far weaker than the Dean drag force. Therefore, the particles will migrate along the Dean vortex, and the inertial focusing effect does not occur. When *R_f_* tends toward ∞, the inertial lift force is far greater than the Dean drag force, and the particles are not affected by the Dean vortex and focus on the same equilibrium position as the straight channel.

Apart from these two circumstances, the particles will focus on different equilibrium positions under the combined action of the inertial lift force and Dean drag force. This new equilibrium position is different from the position in the straight channel, and the number of focusing points is reduced [40]. Finally, what should be mentioned is that *R_f_* is an approximation of the estimated parameters but is not an accurate theoretical value.

#### 2.4.3. Viscous Drag Force

When a particle moves in the fluid, a shear effect is generated on the contact surface, leading to a viscous drag force on the particle. This viscous drag force of spherical particles in a field can be calculated as

(5) $$F_{d}=S\cdot f_{d}=\pi a^{2}f_{d}/4$$

where *S* is the cross-section of particles, and *a* is the diameter of the particle. *f_d_* is the viscous drag coefficient, which is determined by the Reynolds number and the relative velocity of the fluid to the particle. The effect of the viscous drag force in inertial microfluidics will not only drag the particle along the flow direction but will also make the velocity of the particle tend toward the velocity of the flow.

#### 2.4.4. Magnus Lift Force

In a flow field, when the rotational angular momentum *Ω* of a rotating object is not coincident with its velocity vector *U*, there is a lateral force in the direction perpendicular to the plane composed of the rotational angular momentum vector and the translational velocity vector. Under the condition of this lateral force, the trajectory of the object deflects laterally [52]. For a rigid sphere rotating in a fluid, this phenomenon also exists, and a lateral lift force appears under the condition of the transverse pressure difference. This force is called the Magnus lift force, which is expressed as [53]

(6) $$F_{LR}=\frac{1}{8}\pi a^{3}\rho U\times \Omega$$

where *a* is the diameter of the particle, *ρ* is the density of the fluid, *U* is the relative velocity of the particle and fluid, and *Ω* is the rotational angular momentum of the particle.

#### 2.4.5. Saffman Lift Force

A velocity gradient in the fluid will be generated under the effect of the channel wall, which further leads to the spinning of the particle under the effect of shear force. There is an additional drag force that leads to the particle moving behind the fluid. This slip shear will exert a lateral force on the particle and become a shear-gradient lift force, which is also called the Saffman lift force [54]. For a particle in an unbounded shear flow, the expression of the lateral Saffman lift force on a spherical particle can be calculated by an asymptotic expansion method [55]:

(7) $$F_{S}=KVr^{2}\sqrt{\gamma /\upsilon }$$

where *K* is a numerical constant and is usually regarded as 81.2, *γ* is the velocity gradient, *ν* is the viscosity, and *V* is the relative velocity between the fluid and particles. However, compared with the inertial lift force and Dean drag force, the Magnus lift force and Saffman lift force are always ignored because their magnitudes are obviously lower.

In addition to the forces mentioned above, the particles are also affected by other forces including the “van der Waals force”, “electrostatic force”, and “correction fluid resistance” at the same time. These forces are essentially caused by the interaction between molecules, and their ranges of effect are on the nanometer scale. For example, the effect distance of the “van der Waals force” is less than 10 nm, the effect distance of the “electrostatic force” is less than 100 nm, and the effect distance of the “correction fluid resistance” is less than 1 μm. These intermolecular forces can be almost ignored in a microscale channel. Their exact influence on the particles in inertial focusing have not been reported.

A summary of force analyses is presented in Table 1 with the available information reported in recent years.

## 3. Progress in Inertial Microfluidics

### 3.1. Research on Manipulation in Straight Channels

Straight-channel inertial microfluidics can realize the multi-position focusing of particles, by which different kinds of particles can be sorted. Based on the topological structures of channel sections, we can divide straight-channel inertial microfluidics into two categories: straight-rectangular-channel inertial microfluidics and contraction-expansion array channel (CEA channel) inertial microfluidics.

#### 3.1.1. Straight-Rectangular-Channel Inertial Microfluidics

**Figure 6:**
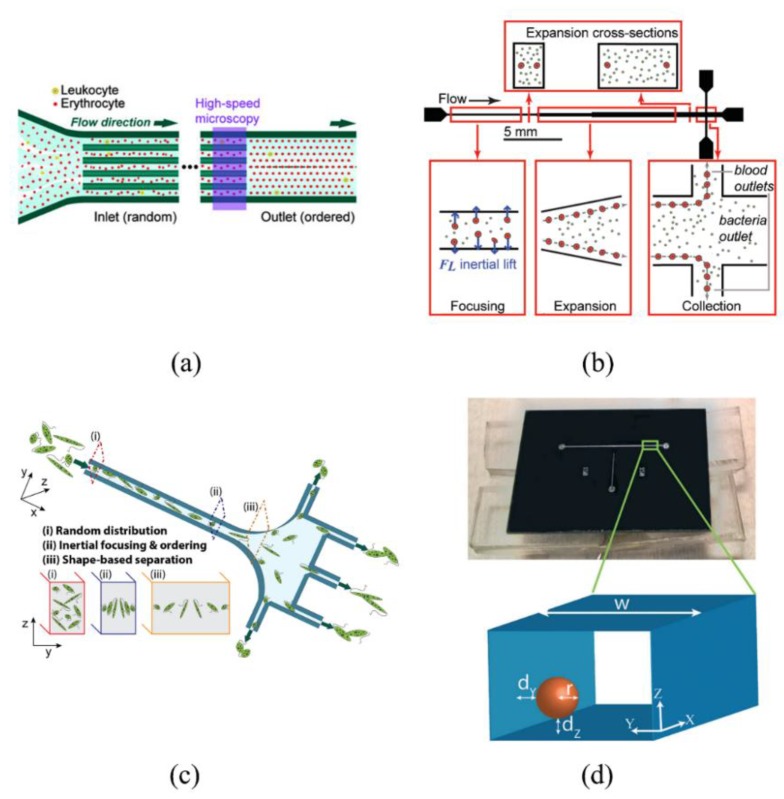
Applications of straight-rectangular-channel inertial microfluidics. (**a**) Counting and differentiation of red blood cells and leukocytes in blood. Ref. [23]. Copyright (2009), with permission from Royal Society of Chemistry; (**b**) Separating pathogenic bacteria cells from diluted blood. Ref. [57]. Copyright (2010), with permission from Wiley Periodicals, Inc.; (**c**) Inertial focusing and ordering of *Euglena gracilis*. Ref. [58]. Copyright (2017), with permission from Springer Nature; (**d**) Inertial manipulation of bubbles in rectangular microfluidics channels. Ref. [60]. Copyright (2018), with permission from Royal Society of Chemistry.

Although it is regarded as a fundamental structure for investigating the mechanism of inertial migration, the straight rectangular channel can also be applied in many fields. Cell separation is the most popular application of inertial microfluidics [56]. In 2009, Di Carlo [23] began to use the focusing principle in a straight rectangular channel to realize the counting and differentiation of red blood cells and leukocytes in the blood. This became an alternative for cell focusing in flow cytometry (Figure 6a). Mach [57] presented a microfluidic device that separates the pathogenic bacteria cells from diluted blood using 40 gradual-expansion single microchannels in a high-throughput, continuous manner. This system can remove 80% of pathogenic bacteria from blood with a throughput of 400 million cells/min (Figure 6b).

Apart from cell separation, the application of a straight rectangular channel is also extended to other biological particles such as bacteria, exsomes, and DNA. Li [58] realized the inertial focusing and ordering of *Euglena gracilis* using a microfluidic device consisting of a straight rectangular microchannel, a gradually expanding region, and five outlets with fluidic resistors to enhance an accurate separation (Figure 6c). Hassan [59] reported on the development of a culture-free bacterial enrichment method to concentrate bacteria from whole blood. This can concentrate bacterial cells and their DNA with a 500-fold reduction in sample volume. Apart from rigid particles manipulation, the manipulation of deformable entities such as bubbles and droplets are considered in inertial microfluidics. Hadikhani [60] showed the trajectory of bubbles flowing in rectangular and square inertial microchannels can be controlled by tuning the balance of forces acting on them with good agreement with the performed numerical simulations (Figure 6d). Edd [61] integrated an oil-and-water clamp device to implement a single-cell drop package with an efficiency 500 times higher than that of a random single package.

In summary, straight-rectangular-channel inertial microfluidics can be used to study particle migration dynamics directly because of its simple structure, and gradually form the basis of the other channel. However, since the length of the channel is long, it is not conducive to miniaturization or integration, which limits its further commercial applications.

#### 3.1.2. Contraction-Expansion-Array-Channel Inertial Microfluidics

Contraction-expansion-array channel (CEA channel) inertial microfluidics [62,63,64] is the deformation of a straight channel through adding or subtracting a specific shape to form a cavity in a straight channel. The expansion structure increases the control flexibility by the section vortex. The vortex is generated at high flow rates. Once the particles reach the expansion region, the wall-effect force diminishes, while the shape of the velocity field decays slowly at downstream distances [65]. In applications, we can divide the CEA channel into symmetrical and asymmetrical channels according to the difference between the contraction-expansion structures.

**Figure 7:**
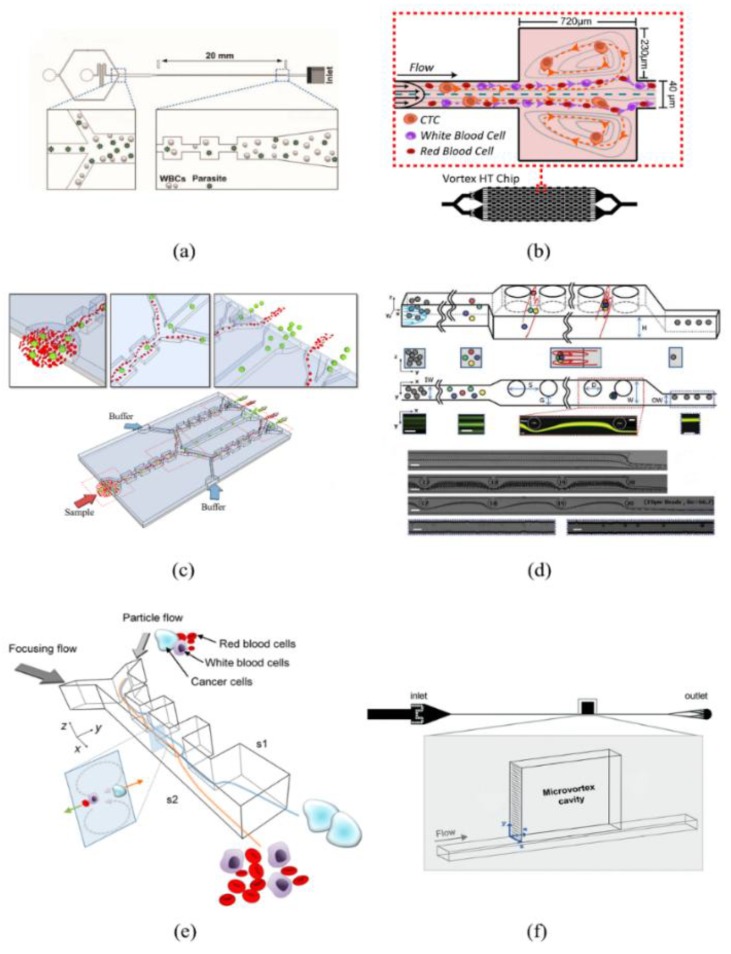
Applications of contraction-expansion-array-channel inertial microfluidics. (**a**) Enriching malaria parasites from blood to facilitate a more reliable and specific PCR-based malaria detection. Ref. [7]. Copyright (2014), with permission from Royal Society of Chemistry; (**b**) High-throughput vortex chip that integrates a crowd of CEA channels to enrich rare circulating tumor cells (CTC). Ref. [66]; (**c**) Multi-stage flow fractionation (MS-MOFF) device designed for separating breast cancer cells from blood. Ref. [67]. Rights managed by AIP Publishing; (**d**) CEA channel simulated by continuous microcolumns to enrich CTCs. Ref. [68]. Copyright (2013), with permission from Royal Society of Chemistry; (**e**) Asymmetric CEA channel used to separate various kinds of cancer cells with high recovery and high throughput. Ref. [69]. Copyright (2013), with permission from American Chemical Society; (**f**) Particle capture and separation along unique particle trajectory made by CEA channel. Ref. [9]. Copyright (2017), with permission from Royal Society of Chemistry.

The symmetrical CEA channel was first reported to realize particle focusing and sorting. Particles with different sizes focused on the wall area near the channel or the central area according to the particle size. Warkiani [7] designed a system utilizing the different focusing behaviors in the CEA channel between blood cells and malaria parasites to enrich malaria parasites from blood, thus facilitating more reliable and specific-PCR-based malaria detection (Figure 7a). Che [66] used a high-throughput vortex chip that integrated a crowd of CEA channels to enrich rare CTCs with high efficiency (83%), a high speed of processing (8 mL/min), and high purity (28.8 ± 23.6 white blood cells per mL of whole blood) (Figure 7b). Moon [67] presented a multi-stage flow fractionation (MS-MOFF) device by combining three single-stage multi-orifice segments designed for separating breast cancer cells from blood. The recovery of breast cancer cells increased from 88.8% to 98.9% through the multi-stage multi-orifice segments (Figure 7c).

The mechanism of the asymmetric CEA channel is approximately the same as that of the symmetrical structure, but the processing difficulty is lower than that of the symmetrical structure. Apart from that, the asymmetric CEA channel can reduce the number of rare samples used in the experiment to avoid waste by using the support of the shear fluid. Chung [68] used continuous microcolumns as a virtual channel to simulate a CEA channel, achieving the same effect as the CEA channel and realizing the separation of circulating tumor cells with a maximum throughput of approximately 1300 particles per second (Figure 7d). Lee [69] characterized inertial particle migration and investigated the migration of blood cells and various cancer cells in a CEA channel, and eventually separated the cancer cells from the whole blood with a recovery rate of 99.1%, blood cell rejection ratio of 88.9%, and throughput of 1.1 × 10^8^ cells/min (Figure 7e). Khojah [9] divided a channel into three stages when he used a CEA channel for particle capture and separation and designed a particle trajectory to complete the separation of cancer cells (Figure 7f). Fan [70] designed a sheathless microfluidic device with trapezoidal CEA channel, which can work over a wide range of Reynolds numbers from 19.1–142.9.

Typical progress of straight-channel inertial microfluidics is presented in Table 2.

In all, as a deformation of a straight rectangular channel, the CEA channel inherits the characteristics of the straight rectangular channel while it expands its application range. In addition, the flexibility is improved by introducing an expansion cavity. However, it is difficult to deal with three kinds of particles or more without the support of the sheath fluid.

### 3.2. Research on the Manipulation in Curved Channels

#### 3.2.1. Arcuate-Channel Inertial Microfluidics

The arcuate channel is the simplest curved channel, and the secondary flow in an arcuate channel is stable. Therefore, it is an ideal model to study the secondary flow in a cross-section and the particle migration characteristics [71,72].

**Figure 8:**
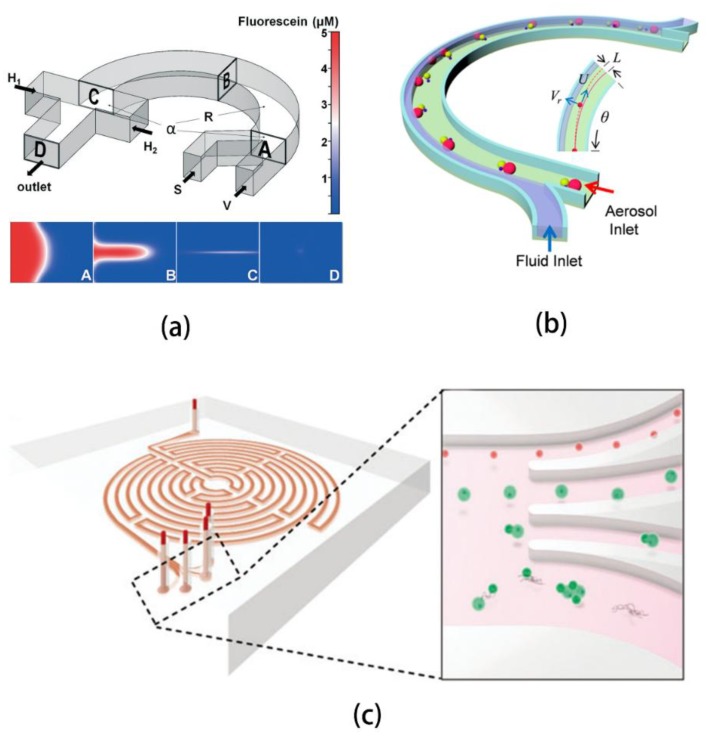
Applications of arcuate channel inertial microfluidics. (**a**) Three-dimensional hydrodynamic focusing device which can serve as a basis for microfluidic flow cytometry. Ref. [73]. Copyright (2013), with permission from Royal Society of Chemistry; (**b**) Continuous airborne microorganism collector for applications in real-time bioaerosol detection. Ref. [74]. Copyright (2017), with permission from American Chemical Society; (**c**) Labyrinth formed by high number of arcuate channels to separate satellite cells and fibroblasts. Ref. [75].

With the development of three-dimensional (3D) fabrication, the applications of the arcuate channel were also extended to the field of 3D channels. 3D particle focusing is a fundamental capability with a wide range of applications such as on-chip flow cytometry. Nawaz [73] designed a 3D hydrodynamic focusing device with different curvature angles, achieving focal positioning along both the lateral and vertical directions on the sub-micrometer scale. This method can serve as a basis for microfluidic flow cytometry with high detection resolution and high performance (Figure 8a). Paie [4] presented a compact microfluidic device capable of 3D particle focusing with high flow rates without external fields or lateral sheath flows. Recently, Choi [74] built a system to sample air and collect liquid to form a stratified flow in a two-phase fluid. These results can be used as a continuous airborne microorganism collector for applications in real-time bioaerosol detection (Figure 8b). Syverud [75] combined a high number of arcuate channels to form a channel structure similar to a labyrinth to achieve the inertial separation of satellite cells and fibroblasts. This unique technology could translate the regenerative potential of satellite cells to engineered tissues for cell therapies (Figure 8c).

In all, the arcuate channel is always used to study the formation and evolution mechanism of a secondary flow and the dynamic mechanism of the migration of particles. This can provide important references for a complex curved channel.

#### 3.2.2. Sinusoidal-Channel Inertial Microfluidics

A sinusoidal channel [76,77] consists of continuous curved channels with alternating curvatures. As different sizes of particles in a sinusoidal channel are focused on the same transverse position, the sinusoidal channel can be used in particle prefocusing devices.

**Figure 9:**
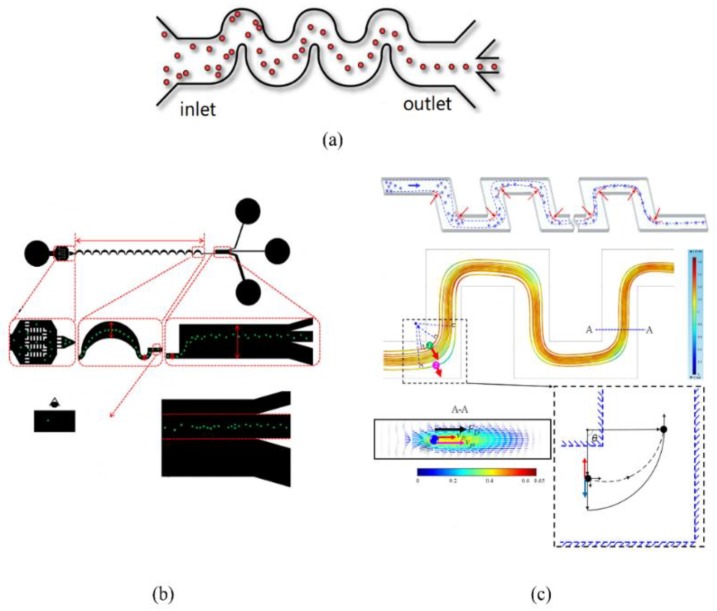
Applications of sinusoidal-channel inertial microfluidics. (**a**) System put forward to separate cells with high throughput of 1 g/h. Ref. [78]; (**b**) Microfluidic concentration for harvesting cyanobacterium Synechocystis sp. PCC 6803. Ref. [80]. Copyright (2017), with permission from Elsevier B.V. All rights reserved; (**c**) Square-wave channel to realize focusing of particles without effects of sheath flow or external force field. Ref. [82]. Copyright (2013), with permission from Springer-Verlag Berlin Heidelberg.

The sinusoidal channel was initially put forward by Di Carlo [78] by increasing the throughput of cell sorting to 1 g/h. Future applications are continuous bioparticle separation, high-throughput cytometry, and large-scale filtration systems (Figure 9a). In addition to cancer cell sorting, application of the sinusoidal channel is also extended to the manipulation of biomolecules. Wang [79] used sinusoidal-channel inertial technology to make 2 μm and even sub-micrometer particles focusing on stable equilibrium positions continuously and accurately. The effect of its chip was demonstrated on cyanobacteria. This study pushed the microfluidic inertial focusing particle range down to the sub-micrometer scale. Wang [80] developed a microfluidic concentration creatively to harvest cyanobacterium Synechocystis sp. PCC 6803. This approach is truly passive and does not require additional reagents, external electronic devices, or fields. The cyanobacteria stream can be steadily separated from the culture medium to obtain a concentrated product. The maximum recovery efficiency achieved was 98.4% in a single microchannel (Figure 9b). Zhang [81] demonstrated the separation of double particles using an inertial microfluidic device based on a simple sinusoidal channel with high efficiency, purity, throughput, and resolution. Apart from the conventional sinusoidal channel, Zhang [82] developed a square-wave channel to realize the focus of particles and demonstrated for the first time that a single focusing streak can be achieved in a symmetric serpentine channel without the effect of the sheath flow or an external force field (Figure 9c).

Owing to various curvatures in a curved channel, a sinusoidal channel can easily increase the throughput through parallel structures, which results in a higher throughput than the arcuate channel or spiral-channel.

#### 3.2.3. Spiral-Channel Inertial Microfluidics

In a curved channel, spiral-channel inertial microfluidics has great potential because of its compact structure and stable flow field. Nowadays, the manipulation method of particles using inertial microfluidics is mostly based on the spiral-channel [83,84]. However, as the curvature of the spiral-channel varies with the position of the particle, the focusing position of the particle can only be obtained by experimental statistics [85,86].

**Figure 10:**
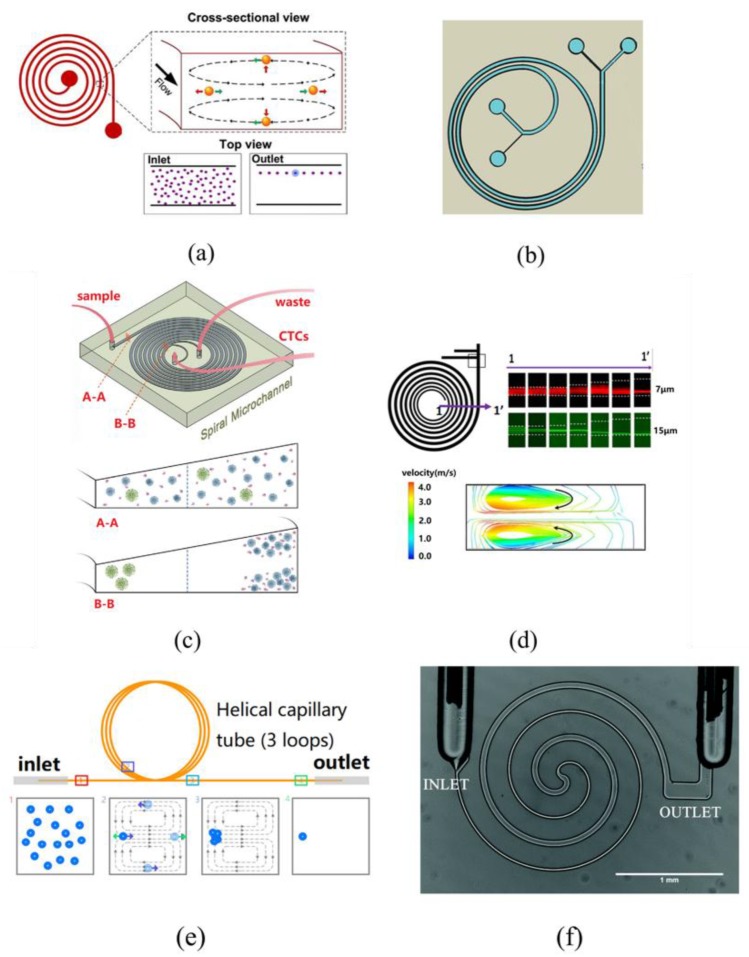
Applications of spiral-channel inertial microfluidics. (**a**) Sheathless flow cytometry system based on principle of Dean-coupled inertial microfluidics. Ref. [88]. Copyright (2009), with permission from Springer Nature; (**b**) Spiral system to realize enrichment of CTCs with recovery of 85% and 99.99% depletion of white blood cells in whole blood. Ref. [90]. Copyright (2015), with permission from Springer Nature; (**c**) Spiral microfluidic device with trapezoidal cross-section. Ref. [94]. Copyright (2017), with permission from Springer Nature; (**d**) Redesign of outlet channel as a series of side-branching channels perpendicular to main channel to separate 7-μm, 10-μm, and 15-μm fluorescent beads. Ref. [97]; (**e**) Three-dimensional spiral inertial focusing device using glass capillary tubes to realize separation of particles with 100% efficiency. Ref. [99]. Rights managed by AIP Publishing; (**f**) Method to isolate bacteria from whole blood rapidly based on principle of Dean flow. Ref. [102].

In spiral-channel inertial microfluidics, the most basic application is high-throughput focusing without a sheath [87]. Bhagat [88] described a sheathless flow cytometry system based on the principle of Dean-coupled inertial microfluidics to focus particles with a high throughput of 2100 particles/s (Figure 10a). Schoeman [89] presented a microfluidic device capable of single-cell encapsulation in droplets by integrating droplet pairing, fusion, and shrinkage. The spiral-channel provided a continuous line of cells and droplets.

Another important application of spiral-channel inertial microfluidics is sorting. In the past several years, the application of spiral microfluidics has mainly focused on the separation of cancer cells after the study of the focusing characteristics of microscale microspheres in spiral-channels. Warkiani [90] designed a spiral system to realize the enrichment of CTCs with a recovery of 85% and 99.99% depletion of white blood cells in whole blood (Figure 10b). On the basis of this structure, Hou [91] and Warkiani [92] realized a series of two structures and a parallel of three structures, respectively. The former combined two spiral structures in series to achieve a higher sensitivity (near 100% detection rate), while the latter paralleled three spiral structures to realize a higher throughput (7.5 mL in less than 10 min). Recently, it was found that compared with a rectangular section, a trapezoid section can change the distribution of the secondary flow, which can widen the distance between the focused particles and the unfocused particles without the effect of a sheath. Abdulla [93] demonstrated simultaneous isolation of different types of CTCs (A549 and MCF-7) from human blood using a cascaded inertial focusing microfluidic channel, consisting of two spiral-channels and one zigzag channel. The recovery of CTCs are 80.75% of A549 and 73.75% of MCF-7, respectively.

To achieve a higher recovery rate and purity, there are different structural designs for the cross-section, inlet, and outlet to improve the properties of inertial-microfluidics channel structures. Kulasinghe [94] evaluated the usage of the spiral microfluidic chip for CTC enrichment and subsequent detection in HNC patients (Figure 10c). Warkiani [95] presented a spiral microfluidic device with a trapezoidal cross-section for ultrafast, label-free enrichment of CTCs with a high separation efficiency of 85%. In a trapezoidal section, the position of the Dean vortex core can be altered to achieve separation. Kwon [96] introduced a cell retention device based on inertial sorting for the perfusion culture of suspended mammalian cells. This device is reliable and clog-free with high recovery (>99%) and cell viability (>97%).

However, the production of a trapezoidal section channel is very expensive since it needs unconventional micromachining. Therefore, it is not suitable for large quantities or low-cost production. The separation principle of spiral-channel inertial microfluidics involves different focusing positions in the channel for particles of different sizes. Thus, with accurate outlet design, we can realize the separation of multi-sized particles. Yousuff [97] designed the outlet as a series of side-branching channels perpendicular to the main channel of egress, demonstrating the collection of up to three particle streams of 7-μm, 10-μm, and 15-μm fluorescent beads with an efficiency exceeding 90% and a throughput of 1.8 mL/min (Figure 10d). Yeh P.Y. [98] used 1-μm and 5.5-μm fluorescent polystyrene microbeads as model particles. These were separated with an efficiency of 99.7% and 98.3%, respectively.

With the development of 3D technology, glass capillary tubes have been widely used in microfluidics. Wang [99] investigated a wide-diameter range of microparticles in a 3D spiral inertial focusing device at various *R_e_* values, and realized the separation with 100% efficiency (Figure 10e). In addition to the single spiral-channel, the double spiral-channel [100] and the gradual expanding channel [101] can be used to separate multi-sized particles.

In recent years, applications have not been limited to cancer cells but have been extended to bacteria, exsomes, and other aspects. Hou [11] described a method to isolate bacteria from whole blood rapidly, based on the principle of the Dean flow to realize the efficient recovery of bacteria, even in low abundance. Cruz [102] designed a structure to reduce the manipulating scale to 1 μm, and validated the focusing effect for Escherichia coli with high throughput (Figure 10f). Choi [103] presented a microfluidic device that uses spiral inertial microfluidics with continuous circulation to separate host cells from viral particles and free nucleic acid.

The typical progress of curved-channel inertial microfluidics is presented in Table 3.

In short, spiral-channel inertial microfluidics is a research hotspot for its compact structure and excellent focusing effect, and has become the best choice for separating biological molecules. However, its mechanism of migration still depends on experimental results and lacks scientific and rigorous theoretical derivation.

### 3.3. Research on Integration of Inertial Microfluidics and Other Technologies

Inertial microfluidics offers an effective approach for particle separation and enrichment without labeling, which is favorable to the following assays. Combining it with protein tracing [104], gene sequencing [105], intracellular drug delivery [106], or high-throughput single-cell analysis [107], we can further investigate the behaviors and the characteristics of some special biomolecules. However, as previously mentioned, owing to the disadvantage of low specificity, people pay more attention to the integration of inertial microfluidics and other active microfluidics to achieve high efficiency, high throughput, and high purity.

**Figure 11:**
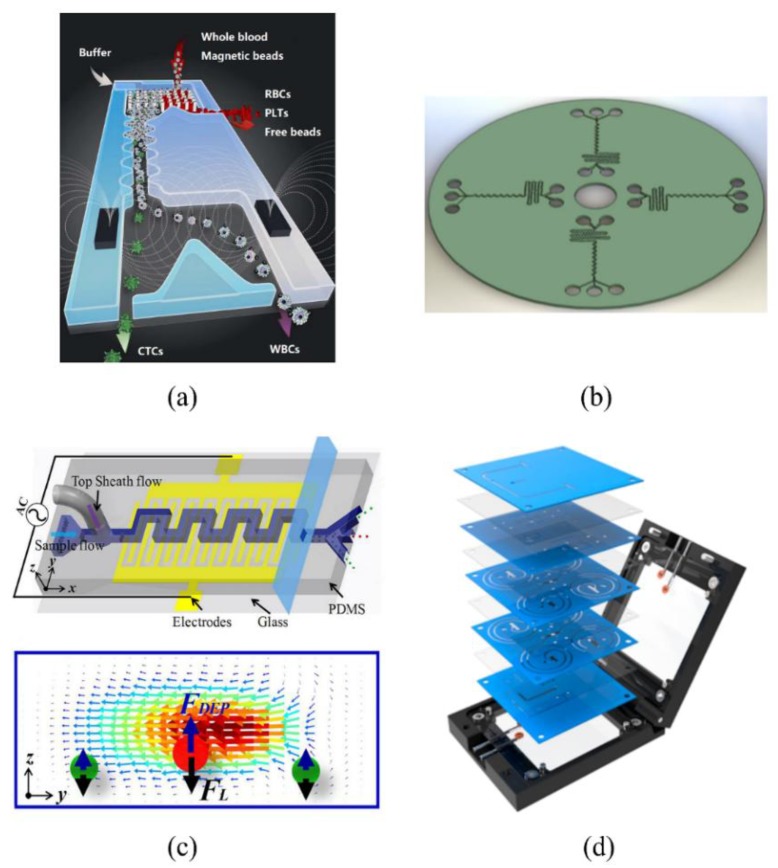
Applications of integration of inertial microfluidics and other active microfluidics. (**a**) Schematic diagram of CTC-iChip, which contains deterministic lateral displacement, inertial focusing in sinusoidal channel, and magnetophoresis sorting. Ref. [113]. Copyright (2014), with permission from Springer Nature; (**b**) Micromixer unit integrated cancer cells and microbeads with high efficiency (97.1%) and inertial flow unit for detection and separation on centrifugal platform. Ref. [116]. Copyright (2014), with permission from Springer Nature; (**c**) An innovative hybrid DEP-inertial microfluidic platform for particle tunable separation. Ref. [111]. Copyright (2018), with permission from American Chemical Society; (**d**) An automated microfluidic instrument with a fully integrated microfluidic device. Ref. [115]. Copyright (2018), with permission from Elsevier B.V.

Among these applications, the CTC-iChip [108] is well known for focusing particles into a straight line. The CTC-iChip uses deterministic lateral displacement, inertial focusing in the sinusoidal channel, and magnetophoresis to sort up to 10^7^ cells/s. The effect of the sinusoidal channel is to line up cells to prepare for precise magnetic separation (Figure 11a). Gerson R. [109] demonstrated an inertial flow unit for the detection and separation in a centrifugal platform and the integration of a magnetic micromixer unit for the first time to create a combination of cancer cells and microbeads. The binding efficiency of cell bead complexes (MCF-7-PS) bound by cancer cells (MCF-7) and microbeads is 97.1% (Figure 11b).

Dielectric technology was also integrated with inertial microfluidics to realize the manipulation of particles. Li [110] integrated dielectric technology with an arcuate channel and finished an in-situ mRNA extraction platform to quantify the marker-gene expression levels of target cells. This integrated platform is a versatile tool for biomedical research. Zhang [111] proposed an innovative hybrid DEP-inertial microfluidic platform for particle tunable separation by introducing an external dielectrophoretic force field and coupling it with inertial forces. In this system, the dimension of the target particle mixture can be varied by adjusting the electrical voltage without redesigning the channel structure or the dimensions (Figure 11c). Liu [112] presented a viscoelasticity-based microfluidic system to directly separate ex-somes from the cell culture media or serum with high separation purity (90%) and recovery (80%).

Since the impact on biological particles is slight, more groups worked on the acoustic methods and integrated them with inertial microfluidics. Li [113] integrated harmless acoustic methods into a straight channel and successfully validated the capability of separating low concentrations of breast cancer cells with a recovery rate better than 83%. Dow [114] integrated a long rectangular inertial channel with acoustic methods to purify bacteria from blood and improved the performance of a bacteriophage-based luminescence assay. To realize the industrialization of inertial microfluidics, Zhang [115] proposed an automated microfluidic instrument with a fully integrated microfluidic device and a set of robust fluid-driven and control units to realize size-based separation of cancer cells in a label-free and high-throughput manner (Figure 11d).

Moreover, a method of circulating tumor cells based on negative immunomagnetics and positive immunomagnetics was also proposed. Stott [116] presented a microvortex-generating herringbone chip to isolate circulating tumor cells with the integration of an immunomagnetic method and straight inertial microfluidics. More valuable scientific discoveries and new devices were achieved by combining these chips with more novel analysis methods. For example, the dynamic capture of CTCs would be achieved through the use of an emulsion hydrogel soft motor proposed by Wang [117]. The cells collected can also be used for cellular analysis and cancer cells prediction based on quantum dot probes [118,119].

A summary of typical progresses in inertial microfluidics integrated with other technologies is presented in Table 4.

## 4. Conclusions and Discussions

The principles and applications of inertial microfluidics can be summarized as follows: (1) When the particles flow in the straight channel, the inertial lift force would be generated, leading to the lateral migration to the dynamic equilibrium position. In the curved channel, because of the Dean vortex, the final equilibrium position of the particle is determined by the combination of inertial lift force and Dean drag force. Particles focusing and separation can be realized mainly due to the inertial lift force, by which high-throughput manipulation can be achieved; (2) The curved channel inertial microfluidics has more advantages to achieve the separation mainly due to the following reasons. Firstly, the curved channel is usually employed to adjust the equilibrium position of the particles which is favorable the collection. Second, the curved channel can contribute to reducing the scale, favorable to further integration on microchip. Third, in the curved channel, the particles with more than two sizes can be separated clearly, which is not easy to realize in straight channels.

Though being widely applied in many fields, there are still many challenges to be solved. Firstly, the principle of the inertial focusing effect has not been deeply studied. The experimental research is often ahead of theoretical exploration and the inertial manipulation in channel with different shapes still lacks the quantitative design rules. Despite the mechanism of particle inertial focusing has been studied extensively, they often focused on the number of focusing equilibrium position and other factors, but few in the mechanical behavior of flow field. To better apply inertial microfluidics, we should focus on exploring the mechanism and hydromechanics characteristics of Dean drag force and its influence on the final particles’ positions. Secondly, the studies of the theoretical model were mainly through rigid particles. However, the research object of inertial microfluidics concentrates on the elastic particles such as cells or vesicles. Therefore, the effect of the deformation should be considered in the theoretical model. Thirdly, as the research object of the inertial microfluidic is extended from micro particles like cells to nanoparticles like DNAs, the previous models may not be applicable due to many factors neglected previously in macro theoretical models. Nanofluidics effects should be taken into consideration, what can extend inertial microfluidics to inertial nanofluidics.

To achieve a wider range of applications, inertial microfluidics is also faced with many problems. First, the observation of particles flowing in a channel is mainly accomplished through experiments but not through CFD technology. However, owing to a lack of mathematical descriptions of physical phenomena, it is difficult to achieve accurate three-dimensional simulations when using only CFD. This needs further detailed studies. Second, a device structure based on inertial microfluidic technology is simple. Thus, design and operation control that depend only on the structure are not simple to determine. Therefore, the manipulation properties can be greatly improved when they are integrated with other active microfluidics such as deterministic lateral displacement and magnetophoresis. Inertial microfluidics can be used as a preprocessing unit and can be integrated with additional detection units to achieve efficient manipulation.

Inertial microfluidics has been approved as an effective approach to particle focusing and separation. More progress is expected regarding theoretical models, simulation tools, and corresponding detection methods. With the trend of device miniaturization, the application of inertial microfluidics can be extended to more areas in biochemical analysis, medical diagnosis, and drug screening.

## Figures and Tables

**Table 1:** Summary of force analyses.

Category	Expression	Cause	Annotations	Reference
lift force				
Inertial lift force	$F_{L}=f_{L}\left(R_{e},x/h\right)\cdot \rho U^{2}a^{4}/H^{2}$	inertial focusing effect	*f_L_*: a nondimensional lift coefficient; *h*: height of the rectangular cross-section; *ρ*: density of fluid; *U*: maximum velocity of fluid; *a*: diameter of particle; *H*: hydraulic diameter;	[41]
Magnus lift force	$F_{LR}=\frac{1}{8}\pi a^{3}\rho U\times \Omega$	when the rotational angular momentum *Ω* of the rotating object is not coincident with its velocity vector *U*, there is a lateral force	*a*: diameter of particle; *ρ*: density of fluid; *U*: relative velocity of the particle and the fluid; *Ω*: rotational angular momentum of particle	[53]
Saffman lift force	$F_{S}=KVr^{2}\sqrt{\gamma /\upsilon }$	A velocity gradient in the fluid will be generated under the effect of channel wall which further leads to the spinning of the particle under the effect of shear force	*K*: numerical constant, usually regarded as 81.2; *V*: relative velocity between the fluid and particles; *γ*: velocity gradient; *ν*: viscosity;	[55]
drag force				
Dean drag force	$F_{Stokes}=3\pi \mu a_{P}U_{D}$	secondary flow	*μ*: fluid viscosity; *a_p_*: diameter of the particle; *U_D_*: Dean flow velocity	[51]
Viscous drag force	$F_{d}=S\cdot f_{d}=\pi a^{2}f_{d}/4$	shear effect on the contact surface between fluid and particles	*S*: cross-section of particles; *a*: diameter of particle; *f_d_*: viscous drag coefficient	[49]

**Table 2:** Typical progress of straight-channel inertial microfluidics.

Category	Targets	Characteristics	Optimal Performances	Reference
Straight Rectangular Channel	RBCs and WBCs	256 high-aspect parallel channels	•Throughput: 1 million cells/s •Sensitivity: 86% •Specificity: 97%	[23]
	bacteria	40 gradual-expansion single microchannels in a radial array with one inlet and two rings of outlets	•Recovery: >80% •Throughput: 400 million cells/min •Label-free operation	[57]
	*Euglena gracilis* (*E. gracilis*)	a straight rectangular microchannel, a gradually expanding region, and five outlets with fluidic resistors	•Throughput: 800 μL/min (~1300 cells/s) •Purity: 96.8%	[58]
Contraction-Expansion-Array (CEA) Channel	malaria parasite	a high-aspect-ratio single-inlet rectangular microchannel patterned with a contraction-expansion array	•Malaria parasite collection yield: 70.9 ± 11.4% •Process 1 mL of lysed blood in 15 min	[7]
	CTCs	a simple microfluidic device with rectangular reservoir array	•Capture efficiency: up to 83% •Processing speed: 8 mL/min of 10× diluted blood •Purity: 28.8 ± 23.6 white blood cells per mL	[66]
	CTCs	a multi-stage multi-orifice flow fractionation (MS-MOFF) device formed by combining three single-stage multi-orifice segments	•Recovery: greater than 98.9% •Flow rate: 126 μL/min	[67]
	cells and particles	an expanded rectangular channel containing asymmetrically located pillars	•Focusing efficiency: 98.33% •Throughput: 13,000 particles/s	[68]
	CTCs (MCF-7, SK-BR-3, and HCC70)	a CEA microchannel with 50-μm-wide and 1200-μm-long contraction regions	•Cancer cell recovery rate: 99.1% •Blood cell rejection ratio: 88.9% •Throughput: 1.1 × 108 cells/min	[69]

**Table 3:** The typical progress of curved-channel inertial microfluidics.

Category	Targets	Characteristics	Optimum Performances	Reference
Arcuate Channel	polystyrene beads	a series of “microfluidic drifting” based 3D hydrodynamic focusing devices	•Throughput: ~2163 particles/s •Focusing performance: standard deviation of the particle position was ±0.45 μm •Coefficient of variation (CV): 2.37%	[73]
	polystyrene-latex (PSL) particles	MicroSampler: microfluidic-based aerosol-into-liquid sampling system	•Aerosol flow rate: 0.6 L/min •Gas to liquid concentration ratio of ∼2 × 10^3^	[74]
	satellite cells	a microfluidic device termed ‘‘Labyrinth’’	•Myogenic purity: 75.5 ± 1.59% •Greater tetanic forces: 143.6 ± 16.9 mN	[75]
Sinusoidal Channel	polystyrene particles & H1650	sinusoidal channel with various curvatures	•Accuracy: >80 nm •No discernable damage to cells mass •Sorting rate : ~1 g/h	[78]
	cyanobacteria	an inlet, a filter region, an asymmetric serpentine channel, an isolation region, and three collection outlets	•Recovery efficiency: 96% •The cost of device is very low (~$0.002 per channel) •Harvest cyanobacteria at initial concentrations of 0.1, 0.01, or even 0.001 vol %	[80]
	particles	the channel consists of a 15.2-mm serpentine section with 15 periods	•Found particle centrifugal force played a significant role in particle focusing	[82]
Spiral-Channel	neutrally buoyant particles (or cells)	a 10-loop spiral microchannel	•Throughput: ∼2100 particles/s •Low variability: CV < 20%	[88]
	CTCs (MCF-7)	a two-loop spiral microchannel with two inlets and two outlets with a radius of ~10 mm	•Recovery: ≥85% •White blood cell depletion: 99.99% •7.5 mL of blood is lysed and resuspended into 3.75 mL of PBS	[111]
	fluorescent beads	a 7-loop spiral microchannel with one inlet and up to 3 outlets	•Collection efficiency: 90% •Flow rate: 1.8 mL/min	[97]
	microbeads	an inertial microfluidic helical capillary device	•Throughput: 13,000 beads/s •Efficiency: ∼100% •Flow rate: 200 μL/min	[99]

**Table 4:** Typical progresses of inertial microfluidics integrated with other technologies.

Category	Target	Characteristic	Optimum Performance	Reference
Inertial Microfluidics & Magnetophoresis	CTCs	a device integrated deterministic lateral displacement, inertial focusing and magnetophoresis	•Processing rate: 8 mL whole blood/h •Throughput: 10^7^ cells/s •Recovery: 97%	[108]
	cancer cell & microbeads	the integration of a micromixer unit and an inertial flow unit for the detection and separation	•Binding efficiency:97.1%	[105]
	CTCs	a microvortex-generating herringbone-chip	•Processing cell density: 386 ± 238 CTCs/mL •Capture efficiency: 91.8 ± 5.2%	[116]
Inertial Microfluidics & Dielectric	mRNAs in single living cells	a filter region and a serpentine-shape single-cell trapping channel	•An average single-cell occupancy of 94 ± 4% •Flow rate: 2 μL/min	[110]
Inertial Microfluidics & Acoustic	CTCs	taSSAW-based high-throughput cell separation device	•Recovery: 83% •Removal rate of WBCs: ∼90% •Throughput: 1.2 mL/h	[113]
	fluorescent particle	a conductive liquid-based focused surface acoustic wave (CL-FSAW) device	•Mixing efficiency: higher than 90% •Flow rate:120 μL/min •Mixing time of the CL-FSAW device: 20 ms	[120]
	bacteria in blood	a main channel of rectangular cross-section and symmetric trifurcating inlets/outlets	•RBCs remove: more than 85% •Yield of spiked bacteria: 40–60%	[49]

## References

[1] Soltani M., Lin J., Forties R.A., Inman J.T., Saraf S.N., Fulbright R.M., Lipson M., Wang M.D. (2014). Nanophotonic trapping for precise manipulation of biomolecular arrays. Nat. Nanotechnol..

[2] Ren D., Xia Y., Wang B., You Z. (2016). Multiplexed analysis for anti-epidermal growth factor receptor tumor cell growth inhibition based on quantum dot probes. Anal. Chem..

[3] Mandal M.K., Yoshimura K., Saha S., Yu Z., Takeda S., Hiraoka K. (2016). Biomolecular analysis and biological tissue diagnostics by electrospray ionization with a metal wire inserted gel-loading tip. Anal. Chem..

[4] Paiè P., Bragheri F., Di Carlo D., Osellame R. (2017). Particle focusing by 3D inertial microfluidics. Microsyst. Nanoeng..

[5] Xiang N., Ni Z., Yi H. (2018). Concentration-controlled particle focusing in spiral elasto-inertial microfluidic devices. Electrophoresis.

[6] Ren D., Xia Y., Wang J., You Z. (2013). Micropatterning of single cell arrays using the PEG-Silane and Biotin–(Strept) Avidin System with photolithography and chemical vapor deposition. Sens. Actuators B Chem..

[7] Warkiani M.E., Tay A.K.P., Khoo B.L., Xiaofeng X., Han J., Lim C.T. (2015). Malaria detection using inertial microfluidics. Lab Chip.

[8] Tu C., Zhou J., Liang Y., Huang B., Fang Y., Liang X., Ye X. (2017). A flexible cell concentrator using inertial focusing. Biomed. Microdevices.

[9] Khojah R., Stoutamore R., Di Carlo D. (2017). Size-tunable microvortex capture of rare cells. Lab Chip.

[10] Meunier A., Hernándezcastro J.A., Turner K., Li K., Veres T., Juncker D. (2016). Combination of mechanical and molecular filtration for enhanced enrichment of circulating tumor cells. Anal. Chem..

[11] Hou H.W., Bhattacharyya R.P., Hung D.T., Han J. (2015). Direct detection and drug-resistance profiling of bacteremias using inertial microfluidics. Lab Chip.

[12] Pang L., Shen S., Ma C., Ma T., Zhang R., Tian C., Zhao L., Liu W., Wang J. (2015). Deformability and size-based cancer cell separation using an integrated microfluidic device. Analyst.

[13] Alshareef M., Metrakos N., Juarez Perez E., Azer F., Yang F., Yang X., Wang G. (2013). Separation of tumor cells with dielectrophoresis-based microfluidic chip. Biomicrofluidics.

[14] Cristofanilli M., Krishnamurthy S., Das C.M., Reuben J.M., Spohn W., Noshari J., Becker F., Gascoyne P.R. (2015). Dielectric cell separation of fine needle aspirates from tumor xenografts. J. Sep. Sci..

[15] Ren D., Wang J., Wang B., You Z. (2016). Probes for biomolecules detection based on RET-enhanced fluorescence polarization. Biosens. Bioelectron..

[16] Murata M., Okamoto Y., Park Y.S., Kaji N., Tokeshi M., Baba Y. (2009). Cell separation by the combination of microfluidics and optical trapping force on a microchip. Anal. Bioanal. Chem..

[17] Choudhry P. (2016). High-Throughput Method for Automated Colony and Cell Counting by Digital Image Analysis Based on Edge Detection. PLoS ONE.

[18] Pantel K., Brakenhoff R.H., Brandt B. (2008). Detection, clinical relevance and specific biological properties of disseminating tumour cells. Nat. Rev. Cancer.

[19] Ren D., Xia Y., You Z. (2013). Multiplexed living cells stained with quantum dot bioprobes for multiplexed detection of single-cell array. J. Biomed. Opt..

[20] Amini H., Lee W., Di Carlo D. (2014). Inertial microfluidic physics. Lab Chip.

[21] Youngren G.K., Acrivos A. (1975). Stokes flow past a particle of arbitrary shape: A numerical method of solution. J. Fluid Mech..

[22] Segre G. (1961). Radial Particle Displacements in Poseuille Flow of Suspensions. Nature.

[23] Hur S.C., Tse H.T.K., Di Carlo D. (2010). Sheathless inertial cell ordering for extreme throughput flow cytometry. Lab Chip.

[24] Yang S.H., Lee D.J., Youn J.R., Song Y.S. (2017). Multiple-line particle focusing under viscoelastic flow in a microfluidic device. Anal. Chem..

[25] Segre G., Silberberg A. (1962). Behaviour of macroscopic rigid spheres in Poiseuille flow Part 1. Determination of local concentration by statistical analysis of particle passages through crossed light beams. J. Fluid Mech..

[26] Bhagat A.A.S., Kuntaegowdanahalli S.S., Papautsky I. (2009). Inertial microfluidics for continuous particle filtration and extraction. Microfluid. Nanofluid..

[27] Gao Y., Magaud P., Baldas L., Lafforgue C., Abbas M., Colin S. (2017). Self-ordered particle trains in inertial microchannel flows. Microfluid. Nanofluid..

[28] Liu C., Hu G., Jiang X., Sun J. (2015). Inertial focusing of spherical particles in rectangular microchannels over a wide range of Reynolds numbers. Lab Chip.

[29] Di Carlo D., Edd J.F., Humphry K.J., Stone H.A., Toner M. (2009). Particle segregation and dynamics in confined flows. Phys. Rev. Lett..

[30] Chun B., Ladd A.J.C. (2006). Inertial migration of neutrally buoyant particles in a square duct: An investigation of multiple equilibrium positions. Phys. Fluids.

[31] Zhou J., Papautsky I. (2013). Fundamentals of inertial focusing in microchannels. Lab Chip.

[32] Moloudi R., Oh S., Yang C., Warkiani M.E., Naing M.W. (2018). Inertial particle focusing dynamics in a trapezoidal straight microchannel: Application to particle filtration. Microfluid. Nanofluid..

[33] Kim J.A., Lee J.R., Je T.J., Jeon E.C., Lee W. (2018). Size-dependent inertial focusing position shift and particle separations in triangular microchannels. Anal. Chem..

[34] Dean W.R. (1927). XVI. Note on the motion of fluid in a curved pipe. Lond. Edinb. Dublin Philos. Mag. J. Sci..

[35] Dean W.R. (1928). LXXII. The stream-line motion of fluid in a curved pipe. Lond. Edinb. Dublin Philos. Mag. J. Sci..

[36] Ookawara S., Higashi R., Street D., Ogawa K. (2004). Feasibility study on concentration of slurry and classification of contained particles by microchannel. Chem. Eng. J..

[37] Garcia M., Pennathur S. (2017). Inertial particle dynamics in the presence of a secondary flow. Phys. Rev. Fluids.

[38] Dinler A., Okumus I. (2018). Inertial particle separation in curved networks: A numerical study. Chem. Eng. Sci..

[39] Xiang N., Zhang X., Dai Q., Cheng J., Chen K., Ni Z. (2016). Fundamentals of elasto-inertial particle focusing in curved microfluidic channels. Lab Chip.

[40] Di Carlo D. (2009). Inertial microfluidics. Lab Chip.

[41] Bhagat A.A.S., Kuntaegowdanahalli S.S., Papautsky I. (2008). Continuous particle separation in spiral microchannels using dean flows and differential migration. Lab Chip.

[42] Nam J., Lim H., Kim D., Jung H., Shin S. (2012). Continuous separation of microparticles in a microfluidic channel via the elasto-inertial effect of non-Newtonian fluid. Lab Chip.

[43] Yang S., Kim J.Y., Lee S.J., Lee S.S., Kim J.M. (2011). Sheathless elasto-inertial particle focusing and continuous separation in a straight rectangular microchannel. Lab Chip.

[44] Lee D.J., Brenner H., Youn J.R., Song Y.S. (2013). Multiplex particle focusing via hydrodynamic force in viscoelastic fluids. Sci. Rep..

[45] Hur S.C., Henderson-MacLennan N.K., McCabe E.R., Di Carlo D. (2011). Deformability-based cell classification and enrichment using inertial microfluidics. Lab Chip.

[46] Ho B.P., Leal L.G. (1974). Inertial migration of rigid spheres in two-dimensional unidirectional flows. J. Fluid Mech..

[47] Asmolov E.S. (1999). The inertial lift on a spherical particle in a plane Poiseuille flow at large channel Reynolds number. J. Fluid Mech..

[48] Schonberg J.A., Hinch E.J. (1989). Inertial migration of a sphere in Poiseuille flow. J. Fluid Mech..

[49] Martel J.M., Toner M. (2014). Inertial focusing in microfluidics. Annu. Rev. Biomed. Eng..

[50] Zhang J., Yan S., Yuan D., Alici G., Nguyen N.T., Warkiani M.E., Li W. (2016). Fundamentals and applications of inertial microfluidics: A review. Lab Chip.

[51] Gossett D.R., Carlo D.D. (2009). Particle focusing mechanisms in curving confined flows. Anal. Chem..

[52] Briggs L.J. (1959). Effect of spin and speed on the lateral deflection (curve) of a baseball; and the Magnus effect for smooth spheres. Am. J. Phys..

[53] Rubinow S.I., Keller J.B. (1961). The transverse force on a spinning sphere moving in a viscous fluid. J. Fluid Mech..

[54] Saffman P.G.T. (1965). The lift on a small sphere in a slow shear flow. J. Fluid Mech..

[55] Bhagat A.A.S., Kuntaegowdanahalli S.S., Papautsky I. (2008). Enhanced particle filtration in straight microchannels using shear-modulated inertial migration. Phys. Fluids.

[56] Tian F., Zhang W., Cai L., Li S., Hu G., Cong Y., Sun J. (2017). Microfluidic co-flow of Newtonian and viscoelastic fluids for high-resolution separation of microparticles. Lab Chip.

[57] Mach A.J., Di Carlo D. (2010). Continuous scalable blood filtration device using inertial microfluidics. Biotechnol. Bioeng..

[58] Li M., Muñoz H.E., Goda K., Di Carlo D. (2017). Shape-based separation of microalga Euglena gracilis using inertial microfluidics. Sci. Rep..

[59] Hassan M.M., Ranzoni A., Cooper M.A. (2018). A nanoparticle-based method for culture-free bacterial DNA enrichment from whole blood. Biosens. Bioelectron..

[60] Hadikhani P., Hashemi S.M.H., Balestra G., Zhu L., Modestino M.A., Gallaire F., Psaltis D. (2018). Inertial manipulation of bubbles in rectangular microfluidic channels. Lab Chip.

[61] Edd J.F., Di Carlo D., Humphry K.J., Köster S., Irimia D., Weitz D.A., Toner M. (2008). Controlled encapsulation of single-cells into monodisperse picolitre drops. Lab Chip.

[62] Chen Q., Li D., Lin J., Wang M., Xuan X. (2017). Simultaneous Separation and Washing of Nonmagnetic Particles in an Inertial Ferrofluid/Water Coflow. Anal. Chem..

[63] Wang X., Zhou J., Papautsky I. (2013). Vortex-aided inertial microfluidic device for continuous particle separation with high size-selectivity, efficiency, and purity. Biomicrofluidics.

[64] Yang D., Leong S., Lei A., Sohn L.L. (2015). High-throughput microfluidic device for rare cell isolation. Proc. SPIE Int. Soc. Opt. Eng..

[65] Paiè P., Che J., Di Carlo D. (2017). Effect of reservoir geometry on vortex trapping of cancer cells. Microfluid. Nanofluid..

[66] Che J., Yu V., Dhar M., Renier C., Matsumoto M., Heirich K., Pegram M.D. (2016). Classification of large circulating tumor cells isolated with ultra-high throughput microfluidic Vortex technology. Oncotarget.

[67] Moon H.S., Kwon K., Hyun K.A., Seok Sim T., Chan Park J., Lee J.G., Jung H.I. (2013). Continual collection and re-separation of circulating tumor cells from blood using multi-stage multi-orifice flow fractionation. Biomicrofluidics.

[68] Chung A.J., Pulido D., Oka J.C., Amini H., Masaeli M., Di Carlo D. (2013). Microstructure-induced helical vortices allow single-stream and long-term inertial focusing. Lab Chip.

[69] Lee M.G., Shin J.H., Bae C.Y., Choi S., Park J.K. (2013). Label-free cancer cell separation from human whole blood using inertial microfluidics at low shear stress. Anal. Chem..

[70] Fan L.L., Yan Q., Zhe J., Zhao L. (2018). Single particle train ordering in microchannel based on inertial and vortex effects. J. Micromech. Microeng..

[71] Nivedita N., Garg N., Lee A.P., Papautsky I. (2017). A high throughput microfluidic platform for size-selective enrichment of cell populations in tissue and blood samples. Analyst.

[72] Zhao Y., Schwemmer F., Zehnle S., von Stetten F., Zengerle R., Paust N. (2015). Centrifugo-pneumatic sedimentation, re-suspension and transport of microparticles. Lab Chip.

[73] Nawaz A.A., Zhang X., Mao X., Rufo J., Lin S.C.S., Guo F., Levine S.J. (2014). Sub-micrometer-precision, three-dimensional (3D) hydrodynamic focusing via “microfluidic drifting”. Lab Chip.

[74] Choi J., Hong S.C., Kim W., Jung J.H. (2017). Highly enriched, controllable, continuous aerosol sampling using inertial microfluidics and its application to real-time detection of airborne bacteria. ACS Sens..

[75] Syverud B.C., Lin E., Nagrath S., Larkin L.M. (2018). Label-Free, High-Throughput Purification of Satellite Cells Using Microfluidic Inertial Separation. Tissue Eng. Part C Methods.

[76] Oakey J., Applegate R.W., Arellano E., Carlo D.D., Graves S.W., Toner M. (2010). Particle focusing in staged inertial microfluidic devices for flow cytometry. Anal. Chem..

[77] Di Carlo D., Edd J.F., Irimia D., Tompkins R.G., Toner M. (2008). Equilibrium separation and filtration of particles using differential inertial focusing. Anal. Chem..

[78] Di Carlo D., Irimia D., Tompkins R.G., Toner M. (2007). Continuous inertial focusing, ordering, and separation of particles in microchannels. Proc. Natl. Acad. Sci. USA.

[79] Wang L., Dandy D.S. (2017). High-Throughput Inertial Focusing of Micrometer-and Sub-Micrometer-Sized Particles Separation. Adv. Sci..

[80] Wang L., Dandy D.S. (2017). A microfluidic concentrator for cyanobacteria harvesting. Algal Res..

[81] Zhang J., Yan S., Sluyter R., Li W., Alici G., Nguyen N.T. (2014). Inertial particle separation by differential equilibrium positions in a symmetrical serpentine micro-channel. Sci. Rep..

[82] Zhang J., Li W., Li M., Alici G., Nguyen N.T. (2014). Particle inertial focusing and its mechanism in a serpentine microchannel. Microfluid. Nanofluid..

[83] Song H., Rosano J.M., Wang Y., Garson C.J., Prabhakarpandian B., Pant K., Lai E. (2017). Spiral-shaped inertial stem cell device for high-throughput enrichment of iPSC-derived neural stem cells. Microfluid. Nanofluid..

[84] Robinson M., Marks H., Hinsdale T., Maitland K., Coté G. (2017). Rapid isolation of blood plasma using a cascaded inertial microfluidic device. Biomicrofluidics.

[85] Son J., Samuel R., Gale B.K., Carrell D.T., Hotaling J.M. (2017). Separation of sperm cells from samples containing high concentrations of white blood cells using a spiral channel. Biomicrofluidics.

[86] Shen S., Tian C., Li T., Xu J., Chen S.W., Tu Q., Wang J. (2017). Spiral microchannel with ordered micro-obstacles for continuous and highly-efficient particle separation. Lab Chip.

[87] Kim T.H., Yoon H.J., Stella P., Nagrath S. (2014). Cascaded spiral microfluidic device for deterministic and high purity continuous separation of circulating tumor cells. Biomicrofluidics.

[88] Bhagat A.A.S., Kuntaegowdanahalli S.S., Kaval N., Seliskar C.J., Papautsky I. (2010). Inertial microfluidics for sheath-less high-throughput flow cytometry. Biomed. Microdevices.

[89] Schoeman R.M., Kemna E.W., Wolbers F., Berg A. (2014). High-throughput deterministic single-cell encapsulation and droplet pairing, fusion, and shrinkage in a single microfluidic device. Electrophoresis.

[90] Warkiani M.E., Khoo B.L., Wu L., Tay A.K.P., Bhagat A.A.S., Han J., Lim C.T. (2016). Ultra-fast, label-free isolation of circulating tumor cells from blood using spiral microfluidics. Nat. Protoc..

[91] Hou H.W., Warkiani M.E., Khoo B.L., Li Z.R., Soo R.A., Tan D.S.W., Lim C.T. (2013). Isolation and retrieval of circulating tumor cells using centrifugal forces. Sci. Rep..

[92] Warkiani M.E., Khoo B.L., Tan D.S.W., Bhagat A.A.S., Lim W.T., Yap Y.S., Lim C.T. (2014). An ultra-high-throughput spiral microfluidic biochip for the enrichment of circulating tumor cells. Analyst.

[93] Abdulla A., Liu W., Gholamipour-Shirazi A., Sun J., Ding X. (2018). High-throughput Isolation of Circulating Tumor Cells Using Cascaded Inertial Focusing Microfluidic Channel. Anal. Chem..

[94] Kulasinghe A., Tran T.H.P., Blick T., O’Byrne K., Thompson E.W., Warkiani M.E., Punyadeera C. (2017). Enrichment of circulating head and neck tumour cells using spiral microfluidic technology. Sci. Rep..

[95] Warkiani M.E., Guan G., Luan K.B., Lee W.C., Bhagat A.A.S., Chaudhuri P.K., Tan D.S., Lim W.T., Lee S.C., Chen P.C. (2014). Slanted spiral microfluidics for the ultra-fast, label-free isolation of circulating tumor cells. Lab Chip.

[96] Kwon T., Prentice H., De Oliveira J., Madziva N., Warkiani M.E., Hamel J.F.P., Han J. (2017). Microfluidic Cell Retention Device for Perfusion of Mammalian Suspension Culture. Sci. Rep..

[97] Mohamed Yousuff C., B Hamid N.H., Kamal Basha I.H., Wei Ho E.T. (2017). Output channel design for collecting closely-spaced particle streams from spiral inertial separation devices. AIP Adv..

[98] Yeh P.Y., Dai Z., Yang X., Bergeron M., Zhang Z., Lin M., Cao X. (2017). An efficient spiral microchannel for continuous small particle separations. Sens. Actuators B Chem..

[99] Wang X., Gao H., Dindic N., Kaval N., Papautsky I. (2017). A low-cost, plug-and-play inertial microfluidic helical capillary device for high-throughput flow cytometry. Biomicrofluidics.

[100] Sun J., Liu C., Li M., Wang J., Xianyu Y., Hu G., Jiang X. (2013). Size-based hydrodynamic rare tumor cell separation in curved microfluidic channels. Biomicrofluidics.

[101] Russom A., Gupta A.K., Nagrath S., Di Carlo D., Edd J.F., Toner M. (2009). Differential inertial focusing of particles in curved low-aspect-ratio microchannels. New J. Phys..

[102] Cruz J., Zadeh S.H., Graells T., Andersson M., Malmström J., Wu Z.G., Hjort K. (2017). High pressure inertial focusing for separating and concentrating bacteria at high throughput. J. Micromech. Microeng..

[103] Choi K., Ryu H., Siddle K.J., Piantadosi A., Freimark L., Park D.J., Han J. (2018). Negative Selection by Spiral Inertial Microfluidics Improves Viral Recovery and Sequencing from Blood. Anal. Chem..

[104] Ren D., Wang J., You Z. (2014). Long-term monitoring of capase-3 activity in living cells based on the FRET probe composed of quantum dot, nanogold and EGF. RSC Adv..

[105] Gulbahce N., Magbanua M.J.M., Chin R., Agarwal M.R., Luo X., Liu J., Hayden D.M., Mao Q., Ciotlos S., Li Z. (2017). Quantitative Whole Genome Sequencing of Circulating Tumor Cells Enables Personalized Combination Therapy of Metastatic Cancer. Cancer Res..

[106] Nguyen C.T.H., Webb R.I., Lamber L.K., Strounina E., Lee E.C., Parat M.O., McGuckin M.A., Popat A., Cabot P.J., Ross B.P. (2017). Bifunctional Succinylated ε-Polylysine-Coated Mesoporous Silica Nanoparticles for pH-Responsive and Intracellular Drug Delivery Targeting the Colon. ACS Appl. Mater. Interfaces.

[107] Ren D., Cui M., Xia Y., You Z. (2012). Micropatterning and its applications in biomedical research. Prog. Biochem. Biophys..

[108] Karabacak N.M., Spuhler P.S., Fachin F., Lim E.J., Pai V., Ozkumur E., Yang J. (2014). Microfluidic, marker-free isolation of circulating tumor cells from blood samples. Nat. Protoc..

[109] Aguirre G.R., Efremov V., Kitsara M., Ducrée J. (2015). Integrated micromixer for incubation and separation of cancer cells on a centrifugal platform using inertial and dean forces. Microfluid. Nanofluid..

[110] Li X., Tao Y., Lee D.H., Wickramasinghe H.K., Lee A.P. (2017). In situ mRNA isolation from a microfluidic single-cell array using an external AFM nanoprobe. Lab Chip.

[111] Zhang J., Yuan D., Zhao Q., Yan S., Tang S.Y., Tan S.H., Li W. (2018). Tunable particle separation in a hybrid dielectrophoresis (DEP)-inertial microfluidic device. Sens. Actuators B Chem..

[112] Liu C., Guo J., Tian F., Yang N., Yan F., Ding Y., Sun J. (2017). Field-free isolation of exosomes from extracellular vesicles by microfluidic viscoelastic flows. ACS Nano.

[113] Li P., Mao Z., Peng Z., Zhou L., Chen Y., Huang P.H., Truica C.I., Drabick J.J., El-Deiry W.S., Dao M. (2015). Acoustic separation of circulating tumor cells. Proc. Natl. Acad. Sci. USA.

[114] Dow P., Kotz K., Gruszka S., Holder J., Fiering J. (2018). Acoustic separation in plastic microfluidics for rapid detection of bacteria in blood using engineered bacteriophage. Lab Chip.

[115] Zhang X., Zhu Z., Xiang N., Long F., Ni Z. (2018). Automated Microfluidic Instrument for Label-Free and High-Throughput Cell Separation. Anal. Chem..

[116] Stott S.L., Hsu C.H., Tsukrov D.I., Yu M., Miyamoto D.T., Waltman B.A., Floyd F.P. (2010). Isolation of circulating tumor cells using a microvortex-generating herringbone-chip. Proc. Natl. Acad. Sci. USA.

[117] Wang H., Liang Y., Gao W., Dong R., Wang C. (2017). An Emulsion-Hydrogel Soft Motor Actuated by Thermal Stimulation. ACS Appl. Mater. Interfaces.

[118] Khoo B.L., Lee S.C., Kumar P., Tan T.Z., Warkiani M.E., Ow S.G.W., Nandi S., Lim C.T., Thiery J.P. (2015). Short-term expansion of breast circulating cancer cells predicts response to anti-cancer therapy. Oncotarget.

[119] Ren D., Wang J., Wang B., You Z. (2017). Quantum dot probes for cellular analysis. Anal. Methods.

[120] Nam J., Jang W.S., Lim C.S. (2018). Micromixing using a conductive liquid-based focused surface acoustic wave (CL-FSAW). Sens. Actuators B Chem..

